# Cutaneous Mucormycosis Following Polytrauma: A Multidisciplinary Approach

**DOI:** 10.7759/cureus.89767

**Published:** 2025-08-11

**Authors:** Harsh Vardhan Baranwal, Vivek K Katiyar, Sumit Sharma, Shashi P Mishra, Satyanam K Bhartiya

**Affiliations:** 1 Department of General Surgery, Institute of Medical Sciences, Banaras Hindu University, Varanasi, IND; 2 Division of Trauma Surgery, Department of General Surgery, Institute of Medical Sciences, Banaras Hindu University, Varanasi, IND

**Keywords:** cutaneous mucormycosis, liposomal amphotericin b, negative-pressure wound therapy (npwt), polytrauma patient, skin graft

## Abstract

Cutaneous mucormycosis is a rare but aggressive fungal infection, increasingly reported in trauma patients without traditional immunosuppressive risk factors. We report the case of a 22-year-old male who developed cutaneous mucormycosis following severe polytrauma from a motorbike-truck collision, presenting with a perineal degloving wound, bilateral testicular avulsion, urethral transection, and subtrochanteric femur fracture. He was resuscitated for hemorrhagic shock and underwent diversion colostomy, suprapubic catheterization, and serial debridement. Persistent necrosis despite antibiotics and negative-pressure wound therapy (NPWT) prompted fungal evaluation, confirming mucormycosis. Intravenous liposomal amphotericin B (5 mg/kg/day) with continued debridement controlled the infection, after which NPWT was reinitiated to optimize the wound bed. Definitive management included split-thickness skin grafting and femoral nailing, alongside testosterone replacement. Antifungal therapy transitioned to oral posaconazole after three weeks of amphotericin B. This case emphasizes early suspicion of mucormycosis in non-healing contaminated wounds, timely antifungal therapy, and staged multidisciplinary management, including cautious NPWT use, to achieve favorable outcomes.

## Introduction

Mucormycosis is an uncommon but aggressive infection caused by angioinvasive fungi of the order Mucorales, particularly *Rhizopus* species. It carries a high mortality rate of approximately 22% for cutaneous presentations and up to 79% for disseminated forms [1]. Though classically seen in immunocompromised individuals (e.g., diabetes, malignancy, neutropenia), cutaneous mucormycosis can develop in immunocompetent hosts following traumatic inoculation via contaminated wounds [2].

Post-traumatic cutaneous mucormycosis is rare yet clinically challenging due to rapid progression to necrosis and poor outcomes if diagnosis is delayed [3]. In trauma-related mucormycosis, prompt surgical debridement is the cornerstone of treatment and significantly improves survival [4]. Systemic antifungal therapy, particularly liposomal amphotericin B, serves as an essential adjunct following aggressive surgical intervention [3,5]. Moreover, step-down therapy with posaconazole is well supported in the management algorithm [5].

Negative-pressure wound therapy (NPWT) has emerged as an effective adjunct in complex wound management by enhancing granulation, reducing bacterial/fungal burden, and promoting tissue healing. However, its use must be carefully timed in mucormycosis, ideally initiated only after microbial clearance to avoid fungal dissemination [6,7].

A multidisciplinary strategy, incorporating trauma surgery, plastic reconstructive surgery, orthopedics, infectious disease, and endocrinology, is vital for optimizing outcomes through coordinated surgical, pharmacologic, and supportive interventions [5].

## Case presentation

A 22-year-old male presented to our level I trauma center following a collision of a motorbike and a truck, with a degloving wound over the perineal region and pain and swelling over the left thigh. On admission, he was hypotensive and required multiple blood transfusions, including four units of packed red blood cells. Primary and secondary surveys were performed as per Advanced Trauma Life Support guidelines, and he responded to initial resuscitation. On examination, he had a Glasgow Coma Scale score of 15/15, a heart rate of 98 beats/minute, a respiratory rate of 20 breaths/minute, blood pressure of 94/52 mmHg, a temperature of 37.8°C, and oxygen saturation of 97% on room air.

On physical examination, a contaminated degloving injury was present with a wound extending 1 cm from the anal verge to the pubic symphysis involving the posteromedial aspect of the left thigh and the shaft of the penis, with a transected and degloved urethra seen outside the penis. Bilateral testes could not be seen or palpated. The left thigh was swollen and tender, and bony crepitus was present in the proximal region. Anal tone was normal, and the digital rectal examination showed fecal staining with no bleeding.

**Figure 1:**
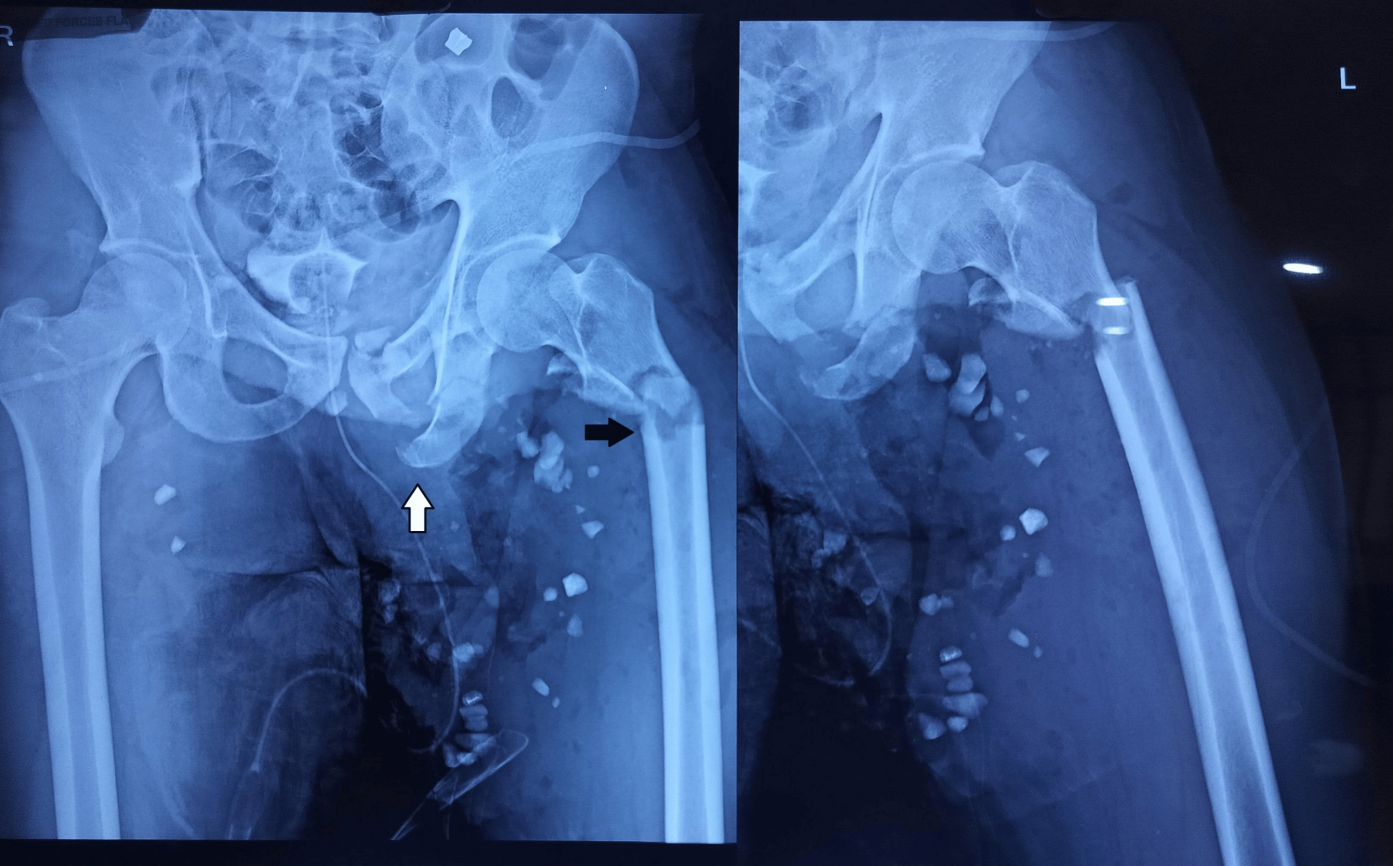
Pelvic X-ray showing a left superior and inferior pubic rami fracture (white arrow), along with a subtrochanteric femur fracture (black arrow). This was managed initially with tibial pin traction.

Blood counts, renal function tests, and imaging studies (including X-rays and a color Doppler for the penis and testicular tissue) were performed. Initial hematological workup revealed a hemoglobin of 7.8 g/dL, an elevated total leucocyte count of 14,100/mm^3^ (reference range: 4,000-11,000/mm^3^), and an elevated C-reactive protein (CRP) of 27.4 mg/L (reference range: <5 mg/L). While the penis had a normal blood flow, neither testis could be visualized on Doppler ultrasonography. X-ray showed left superior and inferior pubic rami as well as a subtrochanteric femur fracture, which was primarily stabilized with tibial pin traction (Figure 1).

**Figure 2:**
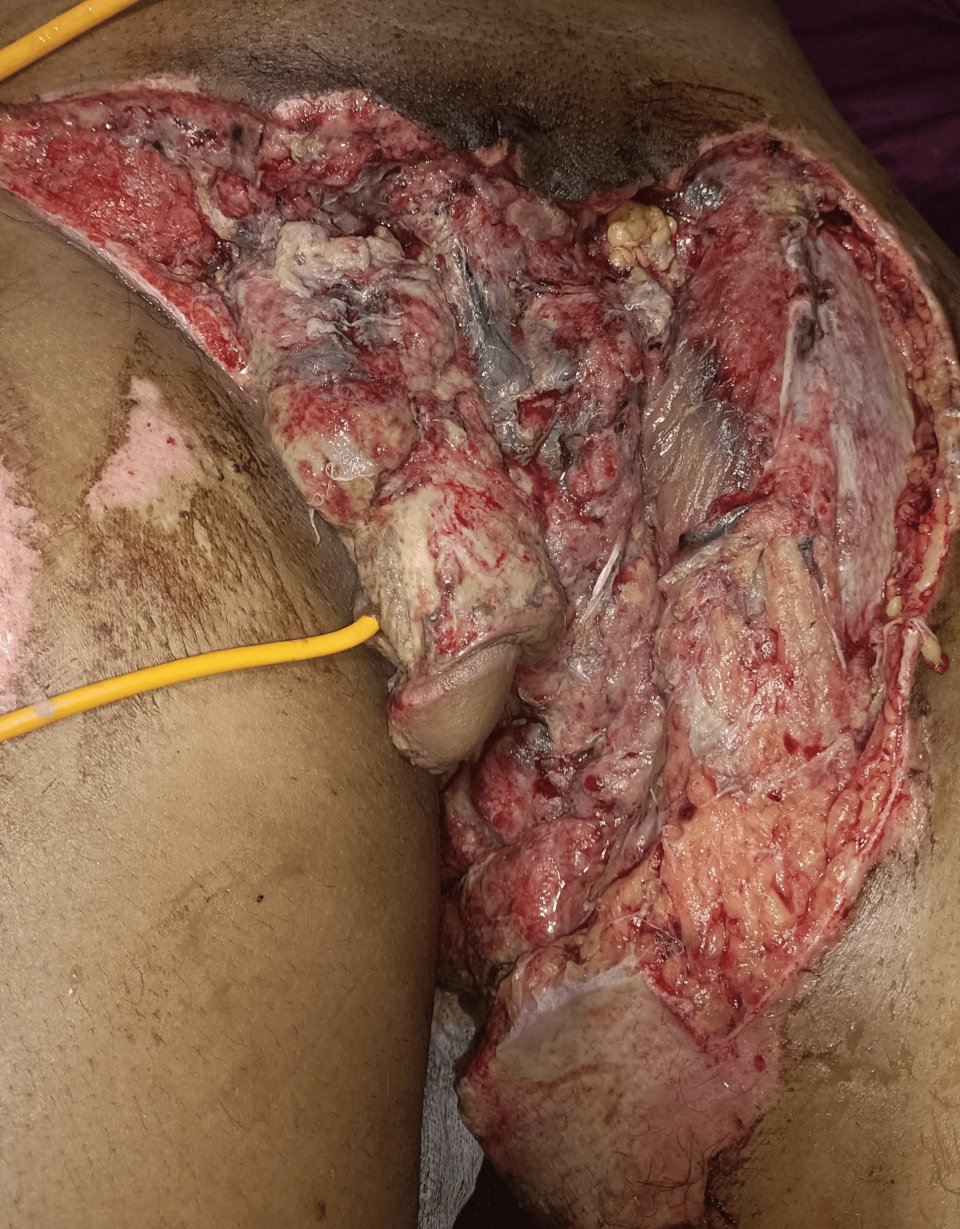
Wound status after initial cleaning and debridement. Perurethral catherization was attempted from the transected urethral segment and was successful (hospital day one).

**Figure 3:**
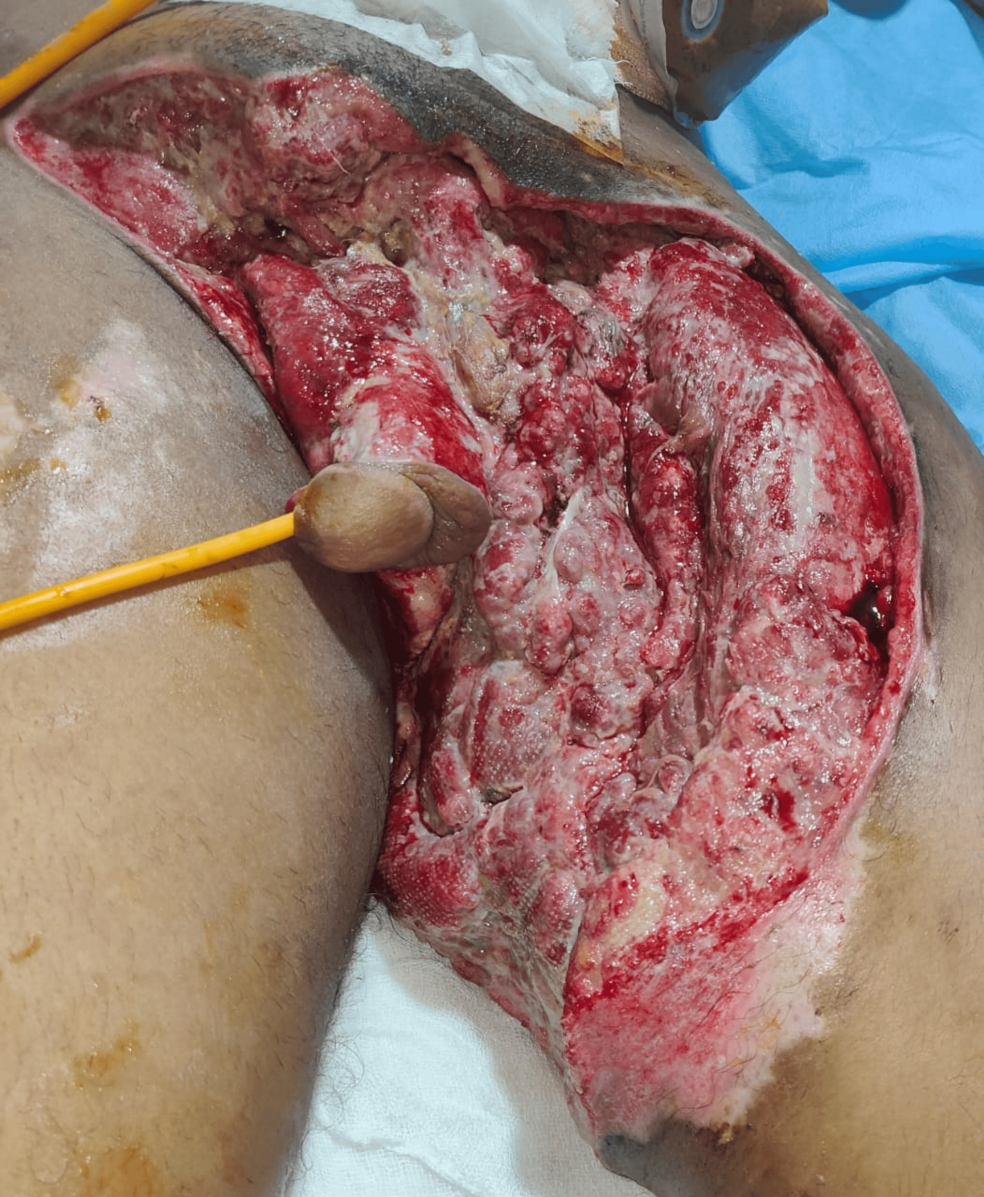
Wound bed after multiple debridement and dressing before the application of negative-pressure wound therapy. The catheter can be seen passing through the meatus (hospital day seven).

**Figure 4:**
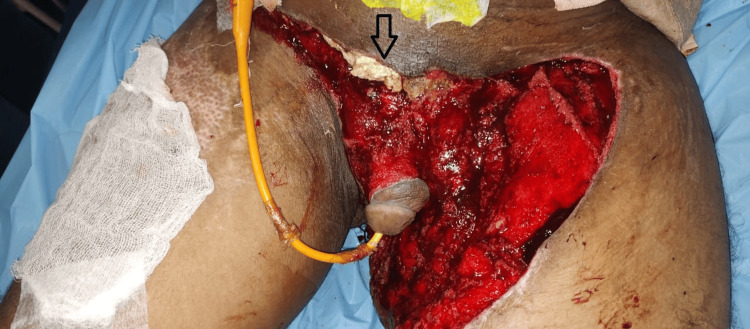
Persistent necrosis seen despite negative-pressure wound therapy (NPWT) and debridement of the wound (black arrow). The rest of the wound showed healthy granulation tissue after NPWT application.

The patient underwent diversion loop sigmoid colostomy with suprapubic catherization with further debridement of the wound (Figure 2). A successful attempt was made to pass a catheter through the penile meatus and the transected urethra in continuum (Figure 3). Serial debridement using mechanical and chemical methods was done over 28 days (Figure 4). Sequential tissue cultures showed *Pseudomonas aeruginosa* sensitive to piperacillin-tazobactam; *Escherichia coli* sensitive to polymyxin B, and *Proteus mirabilis* sensitive to piperacillin-tazobactam and meropenem; *E. coli* sensitive to polymyxin B; and *Acinetobacter baumanii* and *Klebsiella pneumoniae* sensitive to polymyxin B only. The respective antibiotic course was given according to the sensitivity report. One cycle of NPWT was applied once major debridement was complete and healthy granulation tissue was seen. NPWT showed significant seropurulent output of 500 mL on the first day, totaling 3,000 mL over the six-day NPWT course.

**Figure 5:**
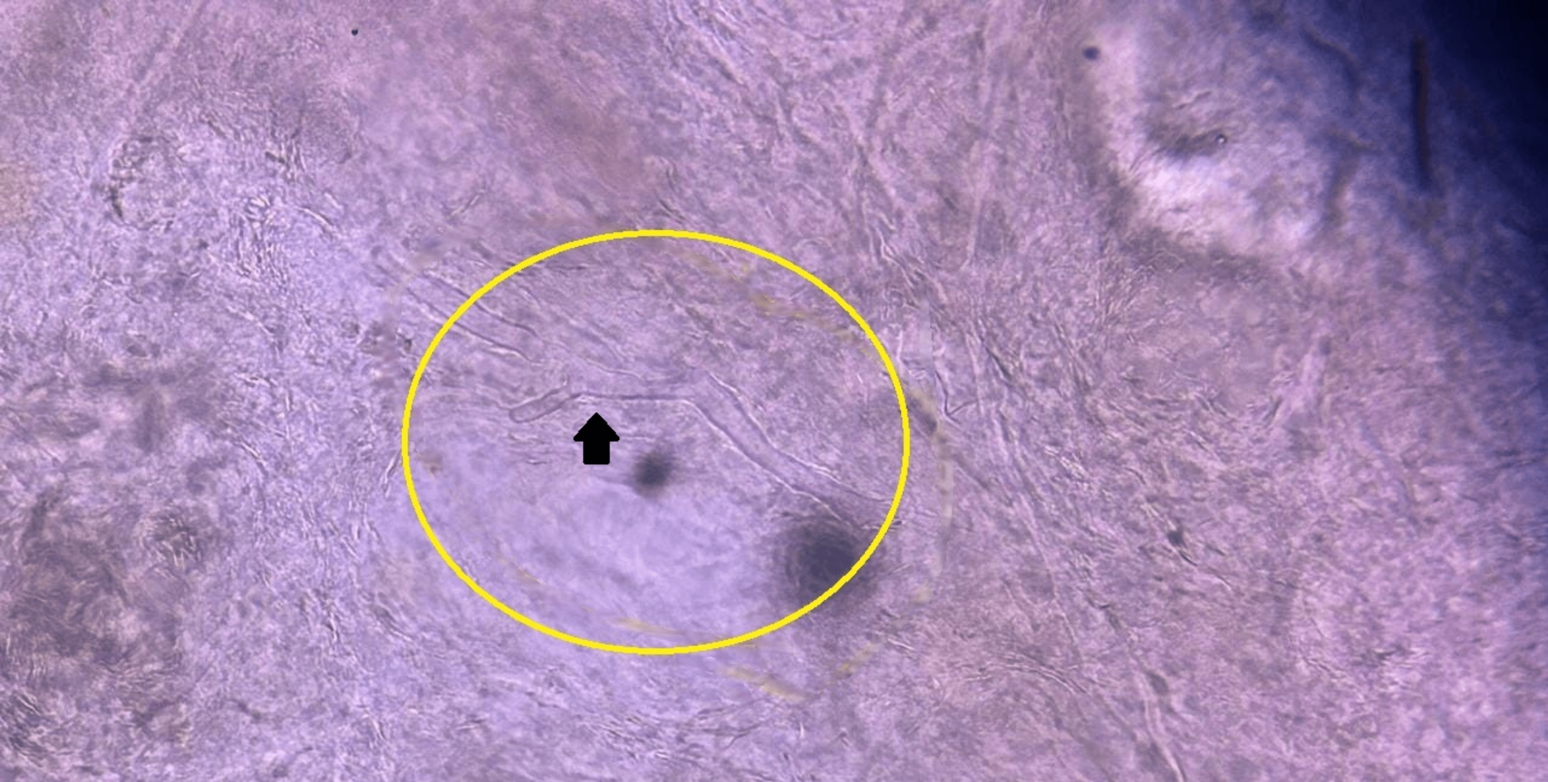
KOH mount of pauci-septate hyphae suggestive of mucormycosis (yellow circle). The black arrow shows minimally broad, ribbon-like hyphae.

Tissue fungal culture was done, given the persistent necrosis. The KOH mount was positive for pauci-septate hyphae, suggestive of cutaneous mucormycosis. Later, the tissue culture was positive for *Rhizopus* spp., confirming the diagnosis of cutaneous mucormycosis (Figure 5).

The patient was shifted to the isolation ward. Liposomal amphotericin B (LAB) (5 mg/kg) was initiated based on the fungal culture result. The patient also received antibiotics targeting bacterial infections (e.g., meropenem and polymyxin B). Treatment continued for two weeks, and renal function monitoring along with serial debridement and dressing was done, with two subsequent mycology reports showing broad aseptate hyphae suggestive of mucormycosis. With negative fungal elements on the next KOH mount, NPWT was applied over the wound. Repeat fungal and bacteriological culture after NPWT was sterile. The patient was shifted to oral posaconazole.

**Figure 6:**
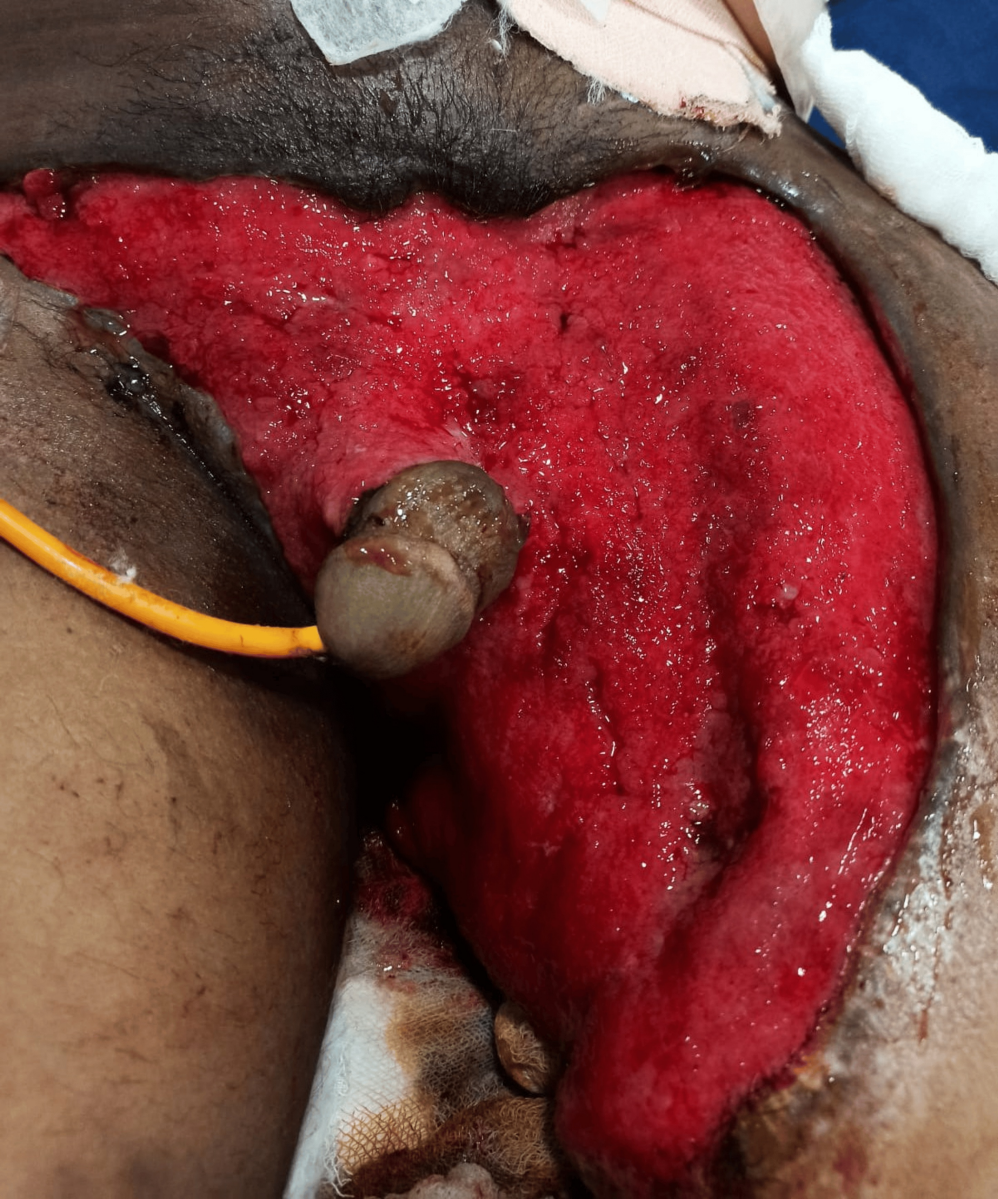
Wound bed after serial debridement and second cycle of negative-pressure wound therapy (NPWT) (hospital day 36). Once the debridement of the necrosed tissue was complete and the second cycle of NPWT was done, significant contraction of wound along with healthy, pink granulation was noted.

**Figure 7:**
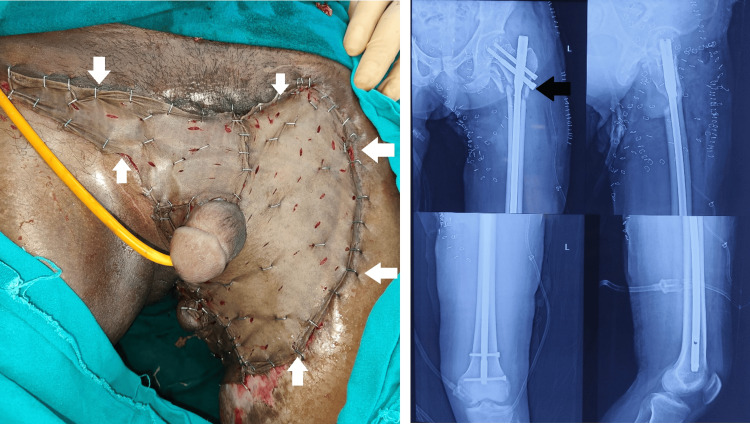
(A) Split-thickness skin graft (white arrows) and (B) intrafemoral nailing (black arrow) done on hospital day 48. The skin graft was taken from the right thigh.

**Figure 8:**
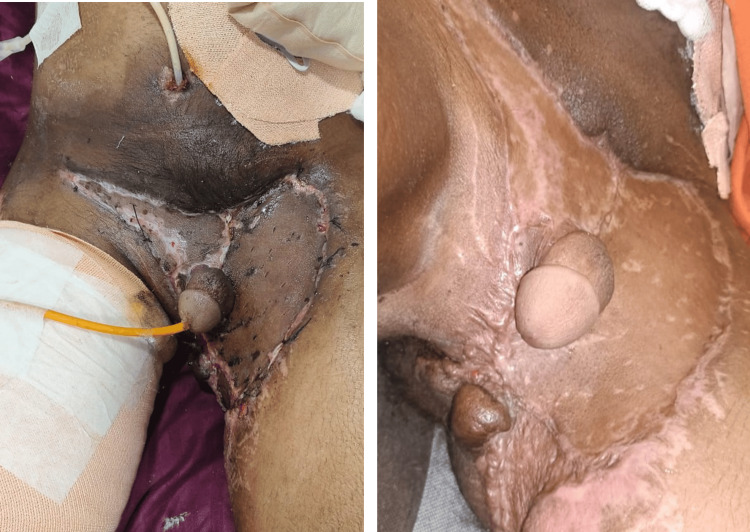
Adequate graft uptake. (A) Hospital day 62. (B) Three-month follow-up in the outpatient department.

After NPWT, the wound showed healthy granulation tissue and was prepared for a split-thickness skin graft (Figure 6). An orthopedic consultation was taken for definitive surgery of the femur fracture. Split-thickness skin graft and proximal femoral nailing were performed under spinal anesthesia in the same setting on hospital day 48 (Figure 7). Endocrinology opinion was taken, given bilateral testes loss and serum testosterone levels of 3.13 ng/dL (reference range: 280-800 ng/dL). Oral testosterone supplementation was started. The graft uptake was satisfactory, and the patient had an uneventful postoperative period (Figure 8). The patient received a total of three weeks of LAB, followed by 10 weeks of posaconazole. He continued to improve and did well on a three-month outpatient department follow-up (Figure 8).

## Discussion

In this post‑traumatic mucormycosis case, the development of aggressive fungal infection, despite intensive surgical care, underscores the virulence of Mucorales, particularly in contaminated wounds of immunocompetent individuals [8,9].

Early and repeated surgical debridement was essential. Trauma-associated mucormycosis frequently requires aggressive and sometimes repeated removal of necrotic tissue to control infection [10]. In one case series, *Mucor circinelloides* cultures only cleared following multiple debridements combined with LAB and topical antifungal therapy alongside NPWT, with resolution achieved by day 37 [11].

Systemic LAB remains the antifungal of choice. It offers effective tissue penetration with a reduced risk of nephrotoxicity compared to conventional formulations, at recommended dosages of 5-10 mg/kg/day, with careful monitoring [11,12]. Oral posaconazole was employed as step-down therapy in our patient, aligning with current practice guidelines [9].

The use of NPWT is nuanced in this setting. While it promotes granulation and wound healing, its application before fungal clearance may facilitate organism spread [13]. In our case, NPWT was paused until serial cultures turned negative, after which it helped prepare the wound bed for definitive closure.

Adjunctive techniques, such as NPWT instillation of amphotericin B, have shown promise in localized cases such as Achilles tendon involvement, achieving infection control and wound stabilization [13]. Although not used in our patient, this method may be considered for future cases in anatomically challenging locations.

Managing post-traumatic mucormycosis requires a coordinated multidisciplinary effort. Trauma surgeons, plastic and orthopedic surgeons, infectious disease specialists, and endocrinologists all contributed to timely surgical intervention, antifungal therapy, wound optimization, fracture fixation, and hormonal replacement, highlighting a model of integrated care.

## Conclusions

The early identification of mucormycosis in trauma patients and the prompt initiation of multimodal therapy are crucial in improving outcomes. NPWT, while effective in wound management, should be carefully applied in the context of fungal infections. This case highlights the importance of a multidisciplinary approach in managing complex trauma wounds, particularly when complicated by rare infections such as mucormycosis. Early surgical debridement, NPWT, and antifungal therapy were key factors in achieving wound healing and successful recovery in this patient.
